# Less physical activity and more varied and disrupted sleep is associated with a less favorable metabolic profile in adolescents

**DOI:** 10.1371/journal.pone.0229114

**Published:** 2020-05-15

**Authors:** Vaka Rognvaldsdottir, Robert J. Brychta, Soffia M. Hrafnkelsdottir, Kong Y. Chen, Sigurbjorn A. Arngrimsson, Erlingur Johannsson, Sigridur L. Guðmundsdottir

**Affiliations:** 1 Center of Sport and Health Sciences, University of Iceland, Reykjavik, Iceland; 2 Diabetes, Endocrinology and Obesity Branch, National Institute of Diabetes and Digestive and Kidney Diseases, Bethesda, MD, United States of America; 3 Department of Sport and Physical Activity, Western Norway University of Applied Sciences, Bergen, Norway; Maastricht University, NETHERLANDS

## Abstract

**Background:**

Sleep and physical activity are modifiable behaviors that play an important role in preventing overweight, obesity, and metabolic health problems. Studies of the association between concurrent objective measures of sleep, physical activity, and metabolic risk factors among adolescents are limited.

**Objective:**

The aim of the study was to examine the association between metabolic risk factors and objectively measured school day physical activity and sleep duration, quality, onset, and variability in adolescents.

**Materials and methods:**

We measured one school week of free-living sleep and physical activity with wrist actigraphy in 252 adolescents (146 girls), aged 15.8±0.3 years. Metabolic risk factors included body mass index, waist circumference, total body and trunk fat percentage, resting blood pressure, and fasting glucose and insulin levels. Multiple linear regression adjusted for sex, parental education, and day length was used to assess associations between metabolic risk factors and sleep and activity parameters.

**Results:**

On average, participants went to bed at 00:22±0.88 hours and slept 6.2±0.7 hours/night, with 0.83±0.36 hours of awakenings/night. However, night-to-night variability in sleep duration was considerable (mean ± interquartile range) 0.75±0.55 hours) and bedtime (0.64±0.53 hours) respectively. Neither average sleep duration nor mean bedtime was associated with any metabolic risk factors. However, greater night-to-night variability in sleep duration and bedtime was associated with higher total body and trunk fat percentage, and less physical activity was associated with higher trunk fat percentage and insulin levels.

**Conclusion:**

Greater nightly variation in sleep duration and in bedtime and less physical activity were associated with a less favorable metabolic profile in adolescents. These findings support the idea that, along with an adequate amount of physical activity, a regular sleep schedule is important for the metabolic health of adolescents.

## Introduction

The prevalence of overweight in the world has nearly tripled from 1975–2016, with over 39% of adults and 18% of children and adolescents being overweight or obese [1]. Greater total body and central adiposity is associated with increased risk of cardio-metabolic comorbidities, such as hypertension and diabetes [2, 3]. Prevalence of metabolic syndrome is high among obese children and adolescents and increases with higher central obesity [4]. Along with diet, sleep and physical activity have been identified as important modifiable risk factors implicated in the development of overweight, obesity, and metabolic health problems [5].

The importance of adequate sleep for health and daily functioning in adolescents is well established [6, 7], although most studies are based on subjective data. Most national and international guidelines focus on recommendations for sleep duration, since prior research has demonstrated that insufficient sleep duration during adolescence is associated with a variety of cognitive, psychological, and health risks, including higher body mass index (BMI) [8–10], greater body fat [11], and increased insulin resistance [12]. However, emerging evidence suggests that sleep quality [13–15] and timing may also affect adolescent cardiometabolic risk factors [7]. For instance, later bedtimes are associated with greater BMI [10, 16], body fat [11] and higher systolic blood pressure in children and adolescents [17]. Markers of irregular sleep schedules, such as high variability in sleep duration or greater shifts in sleep timing and duration on weekends, have also been associated with greater adiposity and abdominal obesity [18, 19] and higher BMI and insulin levels [20] in children and adolescents. Studies also suggest that long-term exposure to a disrupted sleep schedule [21] or low physical activity [22] can increase the risk of developing metabolic syndrome, while higher levels of physical activity in children and adolescents are associated with favorable body mass index, lower adiposity, and better cardio-metabolic health [23, 24]. However, adolescent sleep and physical activity are commonly assessed using self-report [25] which tends to overestimate sleep length and physical activity level, suggesting that adolescents likely sleep less [26] and are less active [27] than previously reported.

In one of the few studies to measure adolescent sleep and body composition with objective measures, He *et*. *al*. (2015) found that more variable sleep patterns, but not shorter sleep duration, was associated with greater central adiposity [18]. However, this study did not include a measure of physical activity, and sleep variability was computed over the entire week. We [28] and others [29, 30] have shown that adolescent sleep patterns are quite different on school nights and non-school nights, thus inclusion of non-school nights likely increases calculated sleep variability. Since studies with simultaneous objective measures of metabolic risk factors and school day sleep and activity are sparse, it is not known whether physical activity and sleep contribute to a better cardiometabolic profile independently.

The aim of the study was to examine associations between metabolic risk factors and concurrent objective measures of free-living sleep and physical activity among Icelandic adolescents. We hypothesized that less activity, shorter sleep duration, poorer sleep quality, and more varied sleep schedules will associate with less favorable cardiometabolic profiles.

## Methods

### Study design and data collection

All students attending the second grade in six of the largest primary schools in Reykjavik, Iceland were invited to participate in a longitudinal cohort studying health, cardiovascular fitness, and physical activity initiated at seven to eight years of age (N = 320, 82% participated) [31]. In April of 2015, all 411 students (age 15–16) enrolled in the 10^th^ grade at the respective schools received an invitation letter to participate, regardless of their participation in earlier waves of the study. Previous participants who had changed schools were excluded. During April-June, one week of concurrent measurement of sleep and activity with wrist actigraphy was introduced [28]. Anthropometry, blood pressure measurements, questionnaires, and wrist accelerometers were administered at the schools. Students were driven to The Icelandic Heart Association for dual-energy X-ray absorptiometry (DXA) scanning and blood sampling.

**Fig 1:**
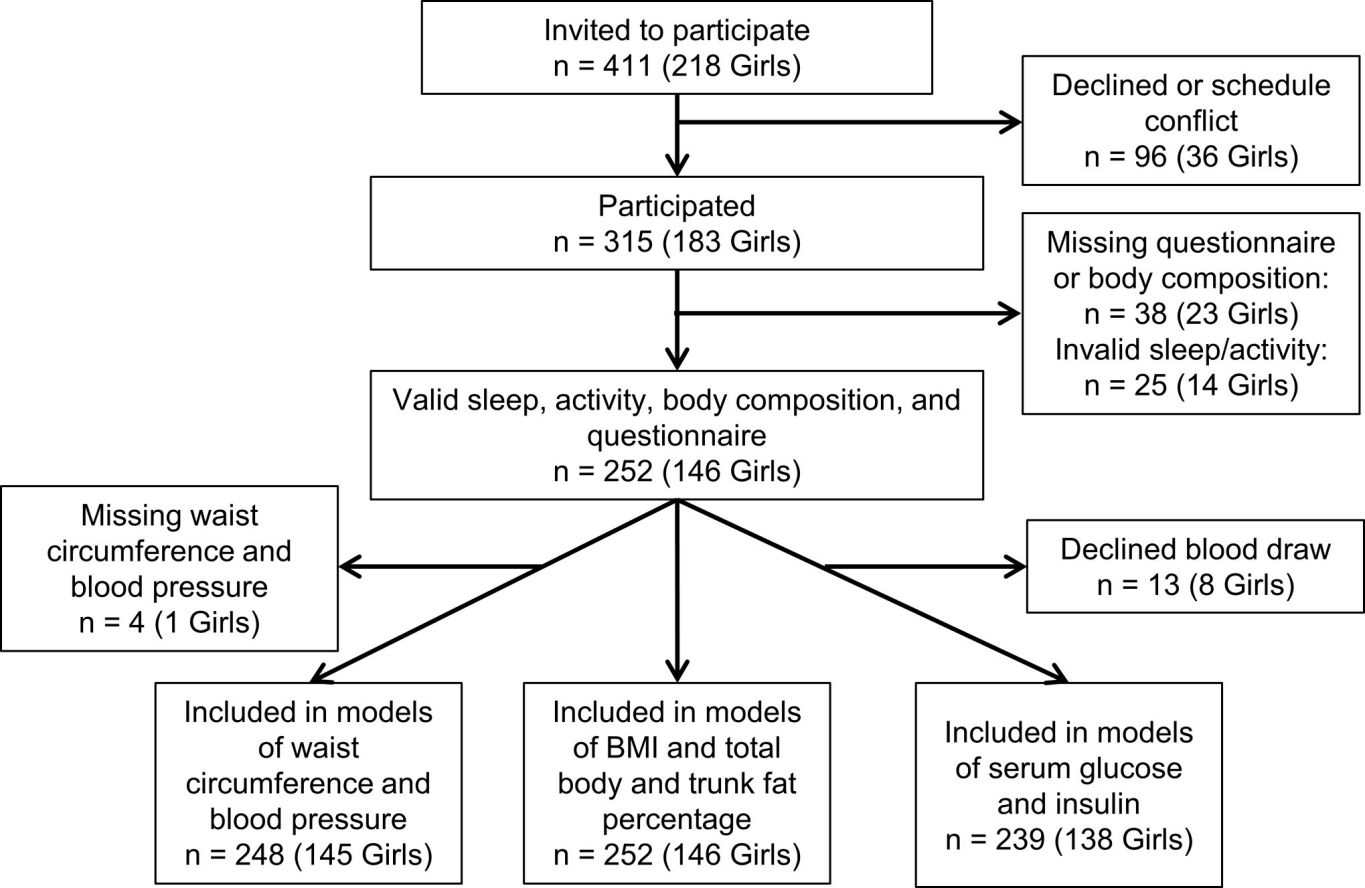
Flow chart describing study participation.

Three hundred and fifteen students agreed to participate (response rate 77%), and 252, or 18.6% of the 15 year-olds living in Reykjavik in 2015 (1355) [32], complete data for questionnaire, body composition, sleep, and physical activity measurements. However, participants with and without complete sleep and activity data did not differ in terms of sex distribution, parental education, age, body composition, or cardiometabolic risk markers (S1 Table). Additionally, two participants were missing waist circumference measurements, 4 did not have valid blood pressure measurements, and 13 refused blood draws for serum glucose and insulin. Study participation is shown in Fig 1.

The current study is an exploratory analysis of the 252 participants with complete data whose sleep and physical activity patterns we have previously reported on in greater detail [28]. Written informed consent was obtained from all participants and their guardians. The study was approved by the National Bioethics Committee, the Icelandic Data Protection Authority (Study number: VSNb2015020013/13.07), and the Icelandic Radiation Safety Authority. The study was conducted in agreement with the guidance provided in the Declaration of Helsinki.

### Sleep and physical activity parameters

Free-living sleep and physical activity were measured with a wrist-worn accelerometer (GT3X+, Actigraph Inc., Pensacola, FL, USA). The accelerometer was placed on the non-dominant wrist at school and the participant was asked to wear it continuously for a week. Physical activity counts and sleep duration, timing, and quality, were computed with Actilife software version 6.13.0 (Actigraph). The Sadeh algorithm, validated for adolescents [33], was used to detect sleep onsets and awakenings, which were visually inspected and adjusted as necessary by two expert scorers based on daily sleep logs maintained during the week of actigraphy. Wear time and vector magnitude of physical activity counts from 12 midnight to 12 midnight the next day [34] were computed in MATLAB (version R2013a MathWorks, Natick, MA, USA) using previously described algorithms [28, 35]. Since only one week of accelerometer data was collected, we focused our analysis on school days (Monday-Friday) and nights (Sunday-Thursday) and did not include data for weekends or holidays. Participants with ≥3 school days and wear time ≥14 hours were included in analyses of sleep. The longest nightly sleep period beginning between 12 noon and 12 noon the next day was used in the analyses. The wear time requirement is in line with a recent systematic review of accelerometer data collection that recommend a minimum wear time of 10 h/day but also noted 24 h assessment for sleep and activity may require longer wear times than studies focused only on waking activity [36]. Sleep and activity parameters are defined in Table 1.

**Table 1:** Sleep and physical activity parameters.

Actigraphy parameter	Definition
Sleep duration	Time spent asleep between sleep onset and awakening (hours/night)
Variability in sleep duration	Night-to-night variation (standard deviation) of sleep duration (hours)
Bedtime	Time of sleep onset (clock time)
Variability in bedtime	Night-to-night variation (standard deviation) of sleep onset (hours)
WASO	Wake time after sleep onset (hours/night)
Physical activity	Activity/wear time (3D-counts/minutes of wear/day)

### Body composition

Height, weight, and waist circumference was measured at participants’ schools. Standing height was measured with a stadiometer (Seca model 217, Seca Ltd. Birmingham, UK) to the nearest 0.1 cm. Body weight was measured to the nearest 0.1 kg using a scale (Seca model 813, Seca Ltd. Birmingham, UK) with participants wearing light clothes. BMI was calculated by dividing weight by height squared (kg/m^2^). Waist circumference was measured to the nearest 0.1 cm using a tape measure next to skin at the narrowest place between the lowest rib and the iliac crest. Fat-free and fat mass were measured with dual-energy X-ray absorptiometry (DXA) using a GE LUNAR scanner (General Electric Lunar iDXA) at the Icelandic Heart Association. All DXA measurements were performed by a certified radiologist. Body fat percentage was calculated by dividing total fat mass by the total body mass (fat mass + lean mass + bone mineral content) and trunk fat percentage was calculated by dividing the total trunk fat mass by total trunk mass. Resting blood pressure was measured on the left arm of seated participants and the average of three measurements was used for analysis.

### Serum measures

Fasting blood samples were obtained using standard procedures after overnight fasting; samples were analyzed for glucose and insulin. Insulin (mU/L) in serum was measured using the INSULIN assay from Roche, a sandwich electrochemiluminescence immunoassay ECLIA on Cobas e 411 (Roche, Switzerland). The inter-assay coefficient of variation was < 5.06% using a frozen serum pool and < 2.36% using quality control samples from Roche. Glucose (mmol/L) in serum was measured using the GLUC2 assay from Roche, an enzymatic reference method with hexokinase. The measurements were done on a Cobas e 311 (Roche, Switzerland). The inter-assay coefficient of variation was <1.65% using a frozen serum pool and <1.66% using quality control samples from Roche.

### Survey questions and environmental data

Students provided the educational attainment of both mother and father from the following options (presented in Icelandic): 1 = “elementary degree”, 2 = “secondary degree”, 3 = “trade school degree”, 4 = “university degree”, 5 = “other”, 6 = “do not know”, 7 = “do not want to answer”. For the current analysis, responses were recoded into a binary variable: 1 = “parent with a university degree” or 0 = “no parent with a university degree”, as described previously [37]. Information on day length (hours of day light) was obtained from National Oceanic and Atmospheric Administration (NOAA) Earth System Research Laboratory Solar Calculator [38]. Information on ethnicity was not collected in this study since the population of Iceland is traditionally ethnically homogenous. For example, during data collection in 2015, only 8% of 15 years old adolescents in Reykjavik were of non-Icelandic origin [39].

### Statistical analyses

T-test for independent samples was used to assess whether participant characteristics, sleep parameters, and physical activity differed between the sexes, unless otherwise noted. Separate multiple linear regression models adjusted for sex, parental education, and day length, were used to explore the associations of each sleep and activity parameter with body composition parameters (BMI, total body fat percentage and trunk fat percentage) and metabolic risk factors (insulin, glucose, blood pressure). In further analysis, body composition and metabolic risk factors were included as response variables while sleep duration, WASO, sleep variability, and physical activity were all simultaneously included as predictor variables in models additionally adjusted for sex, parental education, and day length. In a separate analysis, body composition and metabolic risk factors were again included as response variables while bedtime and bedtime variability were simultaneously included as predictor variables in models additionally adjusted for physical activity, sex, parental education, and day length. Regression analyses were repeated separately by sex for response variables found to be significantly different for boys and girls. Sleep and bedtime variability were log-transformed prior to regression analyses to correct for skewed distributions. Statistical analyses were carried out in Rstudio (Boston, MA, USA, Version 1.1.456) using R statistical software (https://www.r-project.org/, Version 3.5.1). Statistical significance level was set at p<0.05.

## Results

Participant characteristics are shown in Table 2. Although boys were taller and heavier than girls, BMI (overall mean = 21.9±3.0 kg/m^2^) did not differ between the sexes. Overall, 87% of the participants had BMI below 25 kg/m^2^, 10% had 25≤ BMI <30 kg/m^2^, and 2.5% had BMI ≥ 30 kg/m^2^. Boys had lower total body and trunk fat percentage, smaller waist circumference, and slightly higher systolic pressure, but there were no sex differences in age, parental educational attainment, or serum insulin and glucose levels. Participants with and without a parent with a university degree did not differ in characteristics, body composition, blood pressure, or serum insulin and glucose.

**Table 2:** Participants characteristics.

		All (252)	Boys (106)	Girls (146)	p (Boys vs Girls)
Subjects Characteristics					
	Age, years	15.8 ± 0.3	15.8 ± 0.3	15.9 ± 0.3	0.12
	Height, cm	172.0 ± 8.0	178.5 ± 6.0	167.3 ± 5.6	**<0.001**
	Weight, kg	64.8 ± 10.6	68.9 ± 10.3	61.9 ± 9.7	**<0.001**
	Body mass index, kg/m^2^	21.9 ± 3.0	21.6 ± 2.9	22.1 ± 3.1	0.21
	Body fat, %	25.1 ± 8.6	18.2 ± 6.6	30.2 ± 5.9	**<0.001**
	Trunk fat, %	23.6 ± 9.6	17.0 ± 7.8	28.3 ± 7.8	**<0.001**
	Waist circumference, cm*	70.6 ± 7.1	73.5 ± 6.2	68.6 ± 7.1	**<0.001**
	Diastolic pressure, mmHg*	70.9 ± 5.4	70.2 ± 5.6	71.4 ± 5.1	0.21
	Systolic pressure, mmHg*	114.4 ± 9.7	118.6 ± 9.9	111.6 ± 8.3	**<0.001**
	Glucose, mmol/L**	4.9 ± 0.5	4.9 ± 0.4	4.9 ± 0.5	0.57
	Insulin, mU/L**	9.7 ± 4.7	8.4 ± 3.7	10.6 ± 5.2	0.73
	Parent with university degree, N (%)	193 (76.6%)	83 (78.3%)	110 (75.3%)	0.69

Data presented as mean ± standard deviation

*248 participants (103 Boys, 145 Girls)

**239 participants (101 Boys, 138 Girls); Boldface type indicates significant difference (p<0.05).

Sleep and physical activity parameters did not differ between the sexes (Table 3) or between those with or without a parent with a university degree. On average, participants spent 7.05 ± 0.82 hours in bed on school nights, going to bed at 00:22 ± 0.88 hours and rising at 07:27 ± 0.62 hours. While in bed, participants were awake 0.83 ± 0.36 hours and asleep 6.19 ± 0.73 hours. Night-to-night variations in bedtime and sleep duration (median ± interquartile range) were 0.75 ± 0.55 hours and 0.64 ± 0.53 hours, respectively.

**Table 3:** Summary sleep and physical activity parameters.

	All (252)	Boys (106)	Girls (146)	p (Boys Vs. Girls)
Time in bed, hours/night	7.05 ± 0.82	6.99 ± 0.84	7.09 ± 0.80	0.34
Sleep duration, hours/night	6.19 ± 0.73	6.13 ± 0.78	6.24 ± 0.70	0.28
Bedtime, hh:mm ± hours	00:22 ± 0.88	00:28 ± 0.90	00:18 ± 0.87	0.12
Rise time, hh:mm ± hours	07:27 ± 0.62	07:30 ± 0.68	07:24 ± 0.57	0.25
WASO, hours/night	0.83 ± 0.36	0.83 ± 0.35	0.83 ± 0.36	0.98
Variability in sleep duration, hours*	0.75 ± 0.55	0.77 ± 0.53	0.76 ± 0.57	0.48
Variability in bedtime, hours*	0.64 ± 0.53	0.74 ± 0.52	0.61 ± 0.58	0.26
Activity, (counts/min of wear time) x 1000	2.21 ± 0.50	2.24 ± 0.47	2.18 ± 0.52	0.42
Wear time, hours/night	23.76 ± 0.39	23.69 ± 0.50	23.81 ± 0.29	**0.03**
Valid days, days	4.6 ± 0.6	4.5 ± 0.7	4.7 ± 0.5	0.13
Day length, hours/day	17.46 ± 1.87	17.35 ± 1.86	17.54 ± 1.88	0.42

Data presented as mean ± standard deviation unless otherwise noted; p-values are the result of unpaired T-test unless otherwise noted; WASO: wake after sleep onset

*Sleep variability data presented as median ± interquartile range and p-values are results of Mann-Whitney tests due to skewed distributions; Boldface type indicates significant difference (p<0.05).

The association of metabolic risk factors to physical activity and sleep duration, quality, and variability are shown in Table 4. Average sleep duration was not associated with body composition or metabolic parameters. However, the nightly variability of sleep duration was positively associated with both total body and trunk fat percentages. Physical activity was negatively associated with trunk fat percentage and fasting insulin levels. Neither physical activity nor any of the sleep parameters was associated with fasting plasma glucose. WASO, an indicator of sleep quality, was not associated with any metabolic risk factors.

**Table 4:** Association of metabolic risk factors to physical activity and sleep duration, quality, and variability.

		Sleep duration	WASO	Nightly variability in sleep duration	Physical activity
		B [95% CI] (p)	B [95% CI] (p)	B [95% CI] (p)	B [95% CI] (p)
**Body mass index, kg/m**^**2**^					
	Individual model	-0.275 [-0.786, 0.237] (0.3)	-0.889 [-1.932, 0.154] (0.1)	1.164 [-0.293, 2.622] (0.1)	0.543 [-0.210, 1.295] (0.2)
	Combined model	-0.150 [-0.671, 0.371] (0.6)	-0.817 [-1.860, 0.226] (0.1)	1.255 [-0.223, 2.733] (0.1)	0.535 [-0.238, 1.309] (0.2)
**Trunk fat, %**					
	Individual model	-0.435 [-1.767, 0.898] (0.5)	-2.187 [-4.901, 0.527] (0.1)	**4.214 [0.441, 7.987] (0.03)**	**-2.115 [-4.063, -0.167] (0.03)**
	Combined model	-0.535 [-1.879, 0.809] (0.4)	-2.440 [-5.130, 0.249] (0.1)	3.490 [-0.323, 7.302] (0.07)	**-2.178 [-4.172, -0.183] (0.03)**
**Total body fat, %**					
	Individual model	-0.521 [-1.582, 0.539] (0.3)	-1.722 [-3.885, 0.442] (0.1)	**3.587 [0.584, 6.590] (0.02)**	-1.514 [-3.069, 0.041] (0.06)
	Combined model	-0.575 [-1.646, 0.496] (0.3)	-1.898 [-4.040, 0.244] (0.1)	3.000 [-0.037, 6.037] (0.05)	-1.586 [-3.174, 0.003] (0.05)
**Waist circumference, cm**					
	Individual model	-0.169 [-1.324, 0.986] (0.8)	-1.724 [-4.088, 0.640] (0.2)	2.438 [-0.879, 5.755] (0.1)	-0.473 [-2.181, 1.235] (0.6)
	Combined model	-0.112 [-1.295, 1.071] (0.9)	-1.742 [-4.118, 0.634] (0.1)	2.245 [-1.141, 5.631] (0.2)	-0.463 [-2.217, 1.291] (0.6)
**Diastolic pressure, mmHg**					
	Individual model	0.355 [-0.565, 1.275] (0.4)	1.200 [-0.711, 3.111] (0.2)	-0.112 [-2.786, 2.561] (0.9)	-1.078 [-2.432, 0.276] (0.1)
	Combined model	0.228 [-0.716, 1.172] (0.6)	1.091 [-0.828, 3.011] (0.3)	-0.262 [-2.980, 2.455] (0.8)	-0.976 [-2.373, 0.422] (0.2)
**Systolic pressure, mmHg**					
	Individual model	0.450 [-1.104, 2.005] (0.6)	1.305 [-1.928, 4.537] (0.4)	-1.925 [-6.433, 2.582] (0.4)	0.721 [-1.575, 3.016] (0.5)
	Combined model	0.477 [-1.123, 2.077] (0.6)	1.391 [-1.863, 4.645] (0.4)	-1.562 [-6.169, 3.045] (0.5)	0.823 [-1.546, 3.192] (0.5)
**Glucose, mmol/L**					
	Individual model	-0.076 [-0.168, 0.017] (0.1)	-0.018 [-0.208, 0.172] (0.9)	-0.152 [-0.416, 0.111] (0.3)	0.011 [-0.125, 0.147] (0.9)
	Combined model	-0.085 [-0.180, 0.010] (0.1)	-0.016 [-0.206, 0.174] (0.9)	-0.182 [-0.450, 0.087] (0.2)	-0.026 [-0.166, 0.114] (0.7)
**Insulin, mU/L**					
	Individual model	0.254 [-0.552, 1.061] (0.5)	-0.486 [-2.138, 1.166] (0.6)	1.034 [-1.259, 3.328] (0.4)	**-1.901 [-3.060, -0.743] (0.001)**
	Combined model	0.038 [-0.775, 0.851] (0.9)	-0.721 [-2.352, 0.910] (0.4)	0.546 [-1.754, 2.847] (0.6)	**-1.893 [-3.098, -0.689] (0.002)**

Sleep duration is in units of hours/nights; WASO: wake after sleep onset, in hours/night; Variability in sleep duration was log transformed, units are log_10_(hours); Physical activity is in units of (average daily counts/minutes of wear) x 1000; B represent unstandardized regression coefficients; CI: confidence interval; Individual models adjusted for sex, parental education, and day length; Combined models include sleep duration, WASO, nightly variability in sleep duration, physical activity, sex, parental education, and day length; Boldface type indicates significant relationships (p<0.05).

When average sleep duration, WASO, and physical activity were added as covariates, the significant associations between variability in sleep and total and trunk fat percentage did not persist (Table 4, combined model). However, the negative associations between physical activity and trunk fat percentage and fasting insulin levels remained significant, with standardized β values, presented as β [95% confidence intervals], of -0.114 [-0.218, -0.010] and -0.203 [-0.332, -0.074], respectively. When boys and girls were analyzed separately, the negative relationships between physical activity and total body and trunk fat percentage persisted for boys but not girls (S2 Table).

The association of metabolic risk factors to average bedtime and nightly bedtime variability is shown in Table 5. Mean bedtime was not associated with any of the body composition or metabolic parameters after adjusting for sex, parental education, and day length. However, using the same covariates, bedtime variability was positively associated with BMI, waist circumference, total body and trunk fat percentage. All significant relationships persisted when average bedtime and nightly bedtime variability were included in a combined model, adjusted for physical activity, sex, parental education, and day length. Standardized β values for the log-transformed bedtime variability in the significant outcomes, presented as β [95% confidence intervals] were 0.140 [0.014, 0.267] for BMI, 0.137 [0.016, 0.257 for waist circumference,0.060 [0.070, 0.250] for total body fat percentage, and 0.161 [0.060,0.263] for trunk fat percentage. When girls and boys were analyzed separately, the associations between bedtime variability and total body and trunk fat percentage remained significant for girls but not boys (S3 Table). The relationship between bedtime variability and waist circumference was not significant for either sex when analyzed separately.

**Table 5:** Association of metabolic risk factors to average bedtime and nightly variability in bedtime.

		Bedtime	Nightly variability in bedtime
		B [95% CI] (p)	B [95% CI] (p)
**Body mass index, kg/m**^**2**^			
	Individual model	0.222 [-0.213, 0.658] (0.3)	**1.488 [0.209, 2.766] (0.02)**
	Combined model	0.116 [-0.324, 0.557] (0.6)	**1.445 [0.142, 2.748] (0.03)**
**Trunk fat, %**			
	Individual model	0.456 [-0.678, 1.590] (0.4)	**5.489 [2.200, 8.779] (0.001)**
	Combined model	0.143 [-0.985, 1.272] (0.8)	**5.315 [1.975, 8.655] (0.002)**
**Total body fat, %**			
	Individual model	0.481 [-0.422, 1.383] (0.3)	**4.899 [2.292, 7.506] (0.003)**
	Combined model	0.196 [-0.699, 1.092] (0.7)	**4.721 [2.070, 7.372] (0.001)**
**Waist circumference, cm**			
	Individual model	0.070 [-0.917, 1.057] (0.9)	**3.267 [0.393, 6.141] (0.03)**
	Combined model	-0.142 [-1.144, 0.860] (0.8)	**3.332 [0.388, 6.275] (0.03)**
**Diastolic pressure, mmHg**			
	Individual model	-0.184 [-0.973, 0.606] (0.6)	-0.851 [-3.173, 1.470] (0.5)
	Combined model	-0.100 [-0.904, 0.704] (0.8)	-0.840 [-3.204, 1.524] (0.5)
**Systolic pressure, mmHg**			
	Individual model	-0.400 [-1.733, 0.932] (0.6)	-3.495 [-7.394, 0.404] (0.1)
	Combined model	-0.201 [-1.557, 1.156] (0.8)	-3.353 [-7.342, 0.635] (0.1)
**Glucose, mmol/L**			
	Individual model	0.036 [-0.043, 0.115] (0.4)	-0.02 [-0.256, 0.216] (0.9)
	Combined model	0.038 [-0.042, 0.118] (0.4)	-0.039 [-0.279, 0.202] (0.8)
**Insulin, mU/L**			
	Individual model	0.071 [-0.616, 0.758] (0.8)	0.783 [-1.273, 2.839] (0.5)
	Combined model	0.062 [-0.623, 0.747] (0.9)	0.646 [-1.407, 2.698] (0.5)

Bedtime is in units of hours from midnight; Variability in bedtime was log transformed, units are log10(hours); B represent unstandardized regression coefficients; CI: confidence interval; Individual models adjusted for sex, parental education, and day length; Combined models include bedtime, nightly variability in bedtime, physical activity, sex, parental education, and day length; Boldface type indicates significant relationships (p<0.05).

## Discussion

We studied the free-living sleep and physical activity patterns on school days in a sample of 15-year-old Icelandic boys and girls, and, as hypothesized, we found that greater nightly variation in sleep duration and bedtime, and less physical activity was associated with less favorable indicators of metabolic health. Surprisingly, neither mean bedtime nor average sleep duration on school nights was associated with any of the cardiometabolic risk factors measured in our study. These findings support the idea that, along with an adequate amount of physical activity, a regular sleep schedule is important for metabolic health of adolescents.

We found that the participants in our study had considerable nightly variation in sleep duration (0.75 hours) and bedtime (0.64 hours) on school nights and that higher nightly variability in these parameters was related to greater measures of adiposity. These findings are consistent with previous studies of sleep variability and metabolic health, although to date study of this relationship in adolescents has been sparse. He et al. (2015), also found that high variability in sleep duration was associated with greater central adiposity in a group of similarly aged adolescents, even after controlling for food intake [18]. However, there were several notable differences in our regression analyses: (1) we included a measure of physical activity, a well-documented contributor to body composition and metabolic health, and (2) we excluded non-school nights of sleep, when sleep patterns are typically less regular and very different from school night for adolescents [40]. The exclusion of non-school nights from our analysis may partly explain the lower night-to-night variation in sleep duration for our participants compared to He et al. (2015) (0.75 hours vs. 1.2 hours) [18]. Despite these differences, we observed a similar robust association between sleep duration and bedtime variability and adiposity. For instance, our combined regression model indicates that a 30 min increase in variability in nightly sleep duration would lead to a 1.1% increase in trunk fat and a 0.9% increase in total body fat. All else being equal, a participant in the ninetieth percentile of night-to-night sleep variability in our cohort (the ninetieth percentile, with variability of 1.49 hours over the school week) could be expected to have 2.4 percentage points higher body fat than a participant in the lower range of our cohort (the tenth percentile, with variability of 0.33 hours over the school week). Similarly, the upper range of bedtime variability (the ninetieth percentile, with variability of 1.39 hours over school week) could be expected to have 3.4 percentage points higher body fat than those in the lower range of the cohort (the tenth percentile, with variability of 0.27 hours over the school week). The similarity between relationships with variability in sleep duration and bedtime was also not surprising, since the two measures were highly correlated (r = 0.72, p<0.0001). These findings reinforce the idea that adolescents should maintain a regular sleep schedule.

Contrary to our hypothesis, we did not observe a relationship between mean bedtime or average sleep duration and metabolic risk factors. While a number of previous studies have noted a positive association between self-reported short sleep and obesity in adolescents [41–43], some more recent studies find a lack of this relationship while measuring sleep with actigraphy [17, 18]. In agreement with our findings, He et al. (2015) found that high variability in sleep duration, but not mean sleep duration, was associated with greater central adiposity [18]. However, we did not find an association between average bedtime and blood pressure, as demonstrated by Mi, et al. (2019) in a group of mostly younger adolescents (12.4 ± 2.6 y) [17]. Several population-based studies employing self-report sleep measures have observed U-shaped distributions between sleep duration and markers of obesity [42, 43] and metabolic syndrome [44]. These studies benefitted from large sample-populations and broad ranges of body composition and reported sleep duration. We found no evidence for such U-shaped relationships, but our Icelandic cohort was smaller and more homogenous, with a low prevalence (12%) of overweight and obesity and a high prevalence (88%) of short school night sleep [28]. Thus, our results may reflect a more subtle relationship between sleep parameters and body composition than found in studies with larger samples, broader ranges of sleep duration, and greater prevalence of overweight and obesity.

The positive influence of physical activity and the metabolic health of adolescents is well documented [45]. Our finding that wrist-actigraphy measured physical activity was inversely associated with trunk fat percentage and serum insulin levels is largely confirmatory of previous work and consistent with our hypothesis. To put these findings in perspective, all else being equal, one would expect those with a physical activity in the upper range of the cohort (the ninetieth percentile with 2800 counts/min of wear time each day) to have 5.4 mU/L lower fasting insulin and 3.8 percentage points lower trunk fat than those in the lower range of the cohort (the tenth percentile, with1600 counts/min of wear each day). Although these are substantial cross-sectional differences, they are smaller than the changes in these measures observed during aerobic exercise interventions in adolescents [46].

Body composition and systolic blood pressure differed significantly by sex but were largely in line with previous findings in adolescence [47–49]. Considering these differences, we performed additional sex-specific regression analyses for systolic blood pressure, waist circumference, and total body and trunk fat percentage. We did not find any significant sex-specific associations for systolic blood pressure or waist circumference, although the reduced sample-size may have played a role. We did observe that physical activity was more strongly associated with total body and trunk fat for boys, while variability in bedtime was more strongly associated with body composition for girls. Study of larger samples are needed to confirm and explain the sex-based differences in these associations.

The potential causal pathways between irregular sleep patterns and increased body fat are not yet clear. Study of healthy non-overweight children (5–12 years), Burt et al. (2014), found that shorter sleep duration and poor sleep continuity were associated with overeating and other behavior related to obesity risk [50]. Sleep timing, duration, and quality are known to affect regulatory hormones, such as cortisol and growth hormone, as well as appetite regulatory hormones leptin and ghrelin [51]. Thus, high variability in sleep schedule may affect appetite control and contribute to greater adiposity and markers of poorer metabolic health. However, we did not have a measure of food intake and, thus, cannot explore potential relationships between diet, sleep variability, and metabolic health. Additionally, based on our cross-sectional study design, we cannot rule out reverse causality.

A strength of this study is the objective measurement of sleep patterns, physical activity, and body composition. Most previous studies of sleep in this age group have relied on self- or parent-report of typical time in bed or bed- and rise-times. Self-reported measures tend to over-report sleep time [26, 52] and under-report awakenings during sleep [52]. Wrist actigraphy has been validated against laboratory-based polysomnography in this age group and shown to have higher accuracy than self-report for sleep duration [53] and awakenings [54]. DXA is a highly accurate method of classifying body tissues and assessing regional body fat distribution [55–57].

This study has some limitations. The sample size was relatively small (n = 251). However, it represents 18.6% of the 15-year-old population of Reykjavik in 2015 (n = 1355) [32]. This was an exploratory analysis, following up on our previous work [28], and it was not powered to detect a pre-specified outcome. However, our interpretation of the results was not altered by the results of a Benjamini-Hochberg analysis [58] of the 96 comparisons summarized in Tables 4 and 5, using a false discovery rate (Q) of 0.25, since the p-values of all associations noted as significant were below the Benjamini-Hochberg critical p-value of 0.0335. The cross-sectional nature precludes study of the temporal relationships between sleep, physical activity, and metabolic factors. All measurements were collected from spring until early summer, a period of drastic change in day length and weather in Iceland which could affect sleep timing [59] and physical activity level [59, 60]. Earlier analyses of this cohort found no association between sleep duration and day length [28] but a positive association between physical activity and day length [35]. We attempted to mitigate the influence of day length by statistically controlling for it in all regression models. Finally, our sample is mostly lean and racially and ethnically homogeneous, potentially limiting the generalizability of the results to other populations.

## Conclusion

Greater nightly variation in bedtime and sleep duration and less physical activity was associated with higher fat accumulation and higher insulin levels in 15-year-old adolescents, highlighting the importance of physical activity and maintaining a regular sleep schedule. Further research is needed to determine the longitudinal relationship between sleep, physical activity, and metabolic health from adolescence into adulthood.

## Supporting information

S1 Table Comparison of participants with complete and incomplete sleep and activity data. (DOCX)

S2 Table Association of metabolic risk factors to physical activity and sleep duration, quality, and variability for boys and girls. (DOCX)

S3 Table Association of metabolic risk factors to average bedtime and nightly variability in bedtime for boys and girls. (DOCX)

S1 Data (CSV)
