# Supplementary material for: Less physical activity and more varied and disrupted sleep is associated with a less favorable metabolic profile in adolescents
Source: PLoS One. 2020 May 15;15(5):e0229114. doi: 10.1371/journal.pone.0229114 (PMC7228054; doi:10.1371/journal.pone.0229114)
Supplement: S1 Table — (DOCX) [file pone.0229114.s001.docx]

**Table S1. Comparison of participants with complete and incomplete sleep and activity data**

|  | **Incomplete Data** | **Complete Data** | **p-value** | |
| --- | --- | --- | --- | --- |
| Participants, N (% Female) | 25 (56.0%) | 252 (57.9%) | 1.0 | |
| Parent with university degree, N (%) | 17 (68.0%) | 193 (76.6%) | 0.5 | |
| Age, years | 15.9 ± 0.3 | 15.8 ± 0.3 | 0.2 | |
| Height, cm | 171.9 ± 7.7 | 172.0 ± 8.0 | 0.9 | |
| Weight, kg | 66.4 ± 14.6 | 64.8 ± 10.6 | 0.6 | |
| Body mass index, kg/m^2^ | 22.4 ± 4.0 | 21.9 ± 3.0 | 0.6 | |
| Trunk fat, % | 24.6 ± 10.6 | 23.6 ± 9.6 | 0.7 | |
| Total body fat, % | 26.3 ± 9.5 | 25.1 ± 8.6 | 0.6 | |
| Waist circumference, cm* | 71.0 ± 9.8 | 70.6 ± 7.1 | 0.9 | |
| Diastolic pressure, mmHg* | 70.4 ± 5.6 | 70.8 ± 5.5 | 0.7 | |
| Systolic pressure, mmHg* | 114.6 ± 10.0 | 115.1 ± 12.8 | 0.8 | |
| Glucose, mmol/L** | 4.9 ± 0.3 | 4.9 ± 0.5 | 0.7 | |
| Insulin, mU/L** | 10.8 ± 7.4 | 9.7 ± 4.7 | 0.5 | |
| Data presented as mean ± standard deviation unless otherwise noted; *250 participants (105 boys, 145 girls) with complete data; **24 (11 boys, 13 girls) participants with incomplete data, 239 participants (101 boys, 138 girls) with complete data. | | | |  |
