# Supplementary material for: Less physical activity and more varied and disrupted sleep is associated with a less favorable metabolic profile in adolescents
Source: PLoS One. 2020 May 15;15(5):e0229114. doi: 10.1371/journal.pone.0229114 (PMC7228054; doi:10.1371/journal.pone.0229114)
Supplement: S2 Table — (DOCX) [file pone.0229114.s002.docx]

**Table S2. Association of metabolic risk factors to physical activity and sleep duration, quality, and variability for boys and girls.**

|  |  |  | **Sleep duration** | **WASO** | **Nightly variability in sleep duration** | **Physical activity** |
| --- | --- | --- | --- | --- | --- | --- |
|  |  |  | **B [95% CI] (p)** | **B [95% CI] (p)** | **B [95% CI] (p)** | **B [95% CI] (p)** |
| **Trunk fat, %** | | | | | |  |
|  | Boys | |  |  |  |  |
|  |  | Individual model | -0.553 [ -2.532, 1.425] (0.6) | -2.472 [ -6.865, 1.921] (0.3) | 3.709 [ -2.467, 9.884] (0.2) | **-4.647 [ -7.762, -1.533] (0.004)** |
|  |  | Combined model | -1.064 [ -2.995, 0.867] (0.3) | -2.448 [ -6.695, 1.798] (0.3) | 2.116 [ -3.957, 8.189] (0.5) | **-4.708 [ -7.922, -1.494] (0.005)** |
|  | Girls | |  |  |  |  |
|  |  | Individual model | -0.382 [ -2.218, 1.454] (0.7) | -2.208 [ -5.722, 1.307] (0.2) | 4.219 [ -0.686, 9.125] (0.1) | -0.496 [ -3.010, 2.019] (0.7) |
|  |  | Combined model | -0.064 [ -1.985, 1.857] (0.9) | -2.284 [ -5.842, 1.275] (0.2) | 4.036 [ -1.074, 9.146] (0.1) | -0.540 [ -3.150, 2.069] (0.7) |
| **Total body fat, %** | | | | | |  |
|  | Boys | |  |  |  |  |
|  |  | Individual model | -0.487 [ -2.162, 1.188] (0.6) | -2.174 [ -5.891, 1.543] (0.2) | 3.588 [ -1.629, 8.804] (0.2) | **-3.562 [ -6.219, -0.906] (0.009)** |
|  |  | Combined model | -0.877 [ -2.521, 0.767] (0.3) | -2.165 [ -5.781, 1.45] (0.2) | 2.391 [ -2.78, 7.562] (0.4) | **-3.55 [ -6.286, -0.813] (0.012)** |
|  | Girls | |  |  |  |  |
|  |  | Individual model | -0.601 [ -1.988, 0.785] (0.4) | -1.605 [ -4.266, 1.055] (0.2) | 3.289 [ -0.421, 6.999] (0.1) | -0.196 [ -2.100, 1.708] (0.8) |
|  |  | Combined model | -0.353 [ -1.806, 1.099] (0.6) | -1.605 [ -4.296, 1.087] (0.2) | 2.997 [ -0.868, 6.862] (0.1) | -0.301 [ -2.275, 1.672] (0.8) |
| **Waist circumference, cm** | | | | | |  |
|  | Boys | |  |  |  |  |
|  |  | Individual model | -0.293 [ -1.88, 1.294] (0.7) | -2.447 [ -5.973, 1.08] (0.2) | 1.557 [ -3.479, 6.592] (0.5) | -2.428 [ -5.009, 0.153] (0.1) |
|  |  | Combined model | -0.556 [ -2.143, 1.032] (0.5) | -2.342 [ -5.864, 1.18] (0.2) | 0.737 [ -4.305, 5.780] (0.8) | -2.425 [ -5.078, 0.228] (0.1) |
|  | Girls | |  |  |  |  |
|  |  | Individual model | -0.083 [ -1.762, 1.596] (0.9) | -1.291 [ -4.526, 1.945] (0.4) | 2.874 [ -1.668, 7.417] (0.2) | 0.682 [ -1.615, 2.978] (0.6) |
|  |  | Combined model | 0.327 [ -1.442, 2.096] (0.7) | -1.103 [ -4.391, 2.185] (0.5) | 3.206 [ -1.542, 7.955] (0.2) | 0.822 [ -1.580, 3.223] (0.5) |
| **Systolic pressure, mmHg** | | | | | |  |
|  | Boys | |  |  |  |  |
|  |  | Individual model | 0.090 [ -2.425, 2.605] (0.9) | -0.393 [ -6.031, 5.246] (0.89) | 0.604 [ -7.387, 8.595] (0.881) | 1.043 [ -3.111, 5.196] (0.62) |
|  |  | Combined model | 0.191 [ -2.392, 2.775] (0.9) | -0.429 [ -6.159, 5.301] (0.882) | 0.94 [ -7.265, 9.145] (0.821) | 1.177 [ -3.14, 5.493] (0.59) |
|  | Girls | |  |  |  |  |
|  |  | Individual model | 0.762 [ -1.200, 2.723] (0.4) | 2.875 [ -0.971, 6.720] (0.1) | -3.593 [ -8.949, 1.764] (0.2) | 0.213 [ -2.469, 2.896] (0.9) |
|  |  | Combined model | 0.517 [ -1.538, 2.572] (0.6) | 2.952 [ -0.953, 6.857] (0.1) | -3.211 [ -8.770, 2.349] (0.3) | 0.491 [ -2.295, 3.277] (0.7) |
| Sleep duration is in units of hours/nights; WASO: wake after sleep onset, in hours/night; Variability in sleep duration was log transformed, units are log_10_(hours); Physical activity is in units of (average daily counts/minutes of wear) x 1000; B represent unstandardized regression coefficients; CI: confidence interval; Individual models adjusted for sex, parental education, and day length; Combined models include sleep duration, WASO, nightly variability in sleep duration, physical activity, sex, parental education, and day length; Boldface type indicates significant relationships (p<0.05). | | | | | | |
