# Supplementary material for: Less physical activity and more varied and disrupted sleep is associated with a less favorable metabolic profile in adolescents
Source: PLoS One. 2020 May 15;15(5):e0229114. doi: 10.1371/journal.pone.0229114 (PMC7228054; doi:10.1371/journal.pone.0229114)
Supplement: S3 Table — (DOCX) [file pone.0229114.s003.docx]

**Table S3. Association of metabolic risk factors to average bedtime and nightly variability in bedtime for boys and girls.**

|  |  |  | **Bedtime** | **Nightly variability in bedtime** |
| --- | --- | --- | --- | --- |
|  |  |  | **B [95% CI] (p)** | **B [95% CI] (p)** |
| **Trunk fat, %** | | | | |
|  | Boys | |  |  |
|  |  | Individual model | 0.772 [ -0.996, 2.54] (0.4) | 4.298 [ -1.048, 9.644] (0.1) |
|  |  | Combined model | 0.726 [ -0.995, 2.447] (0.4) | 3.087 [ -2.196, 8.369] (0.2) |
|  | Girls | |  |  |
|  |  | Individual model | 0.339 [ -1.171, 1.849] (0.7) | **6.028 [ 1.703, 10.354] (0.007)** |
|  |  | Combined model | -0.22 [ -1.757, 1.317] (0.8) | **6.249 [ 1.729, 10.768] (0.007)** |
| **Total body fat, %** | | | | |
|  | Boys | |  |  |
|  |  | Individual model | 0.703 [ -0.793, 2.199] (0.4) | 4.279 [ -0.225, 8.783] (0.1) |
|  |  | Combined model | 0.621 [ -0.841, 2.082] (0.4) | 3.335 [ -1.151, 7.82] (0.1) |
|  | Girls | |  |  |
|  |  | Individual model | 0.423 [ -0.718, 1.565] (0.5) | **5.070 [ 1.816, 8.323] (0.002)** |
|  |  | Combined model | -0.039 [ -1.195, 1.118] (0.9) | **5.123 [ 1.722, 8.525] (0.003)** |
| **Waist circumference, cm** | | | | |
|  | Boys | |  |  |
|  |  | Individual model | 0.481 [ -0.949, 1.911] (0.5) | 2.519 [ -1.756, 6.795] (0.2) |
|  |  | Combined model | 0.490 [ -0.945, 1.924] (0.5) | 1.850 [ -2.477, 6.177] (0.4) |
|  | Girls | |  |  |
|  |  | Individual model | -0.197 [ -1.58, 1.186] (0.8) | 3.760 [ -0.251, 7.771] (0.1) |
|  |  | Combined model | -0.590 [ -2.015, 0.835] (0.4) | 4.183 [ -0.002, 8.368] (0.05) |
| **Systolic pressure, mmHg** | | | | |
|  | Boys | |  |  |
|  |  | Individual model | -0.328 [ -2.598, 1.941] (0.8) | -3.382 [ -10.168, 3.404] (0.3) |
|  |  | Combined model | -0.214 [ -2.529, 2.100] (0.9) | -3.126 [ -10.106, 3.855] (0.4) |
|  | Girls | |  |  |
|  |  | Individual model | -0.587 [ -2.213, 1.04] (0.5) | -3.192 [ -7.938, 1.555] (0.2) |
|  |  | Combined model | -0.326 [ -2.015, 1.364] (0.7) | -2.964 [ -7.919, 1.991] (0.2) |
| Sleep duration is in units of hours/nights; WASO: wake after sleep onset, in hours/night; Variability in sleep duration was log transformed, units are log_10_(hours); Physical activity is in units of (average daily counts/minutes of wear) x 1000; B represent unstandardized regression coefficients; CI: confidence interval; Individual models adjusted for sex, parental education, and day length; Combined models include sleep duration, WASO, nightly variability in sleep duration, physical activity, sex, parental education, and day length; Boldface type indicates significant relationships (p<0.05). | | | | |
